# Assessing Sarcopenia in Advanced‐Stage Ovarian Cancer Patients Undergoing Neoadjuvant Chemotherapy: A Case Series

**DOI:** 10.1002/cnr2.2155

**Published:** 2024-08-08

**Authors:** Christelle Schofield, Dennis R. Taaffe, Robert U. Newton, Daniel A. Galvão, Paul A. Cohen, Jin‐Soo Kim, Tarek Meniawy, Carolyn Peddle‐McIntyre

**Affiliations:** ^1^ Exercise Medicine Research Institute Edith Cowan University Joondalup Western Australia Australia; ^2^ School of Medical and Health Sciences Edith Cowan University Joondalup Western Australia Australia; ^3^ School of Medicine University of Western Australia Crawley Western Australia Australia; ^4^ St John of God Hospital Subiaco Western Australia Australia; ^5^ Sir Charles Gairdner Hospital Nedlands Western Australia Australia

**Keywords:** muscle mass, muscle strength, ovarian cancer, sarcopenia

## Abstract

**Objectives:**

In ovarian and other cancers, low muscle mass and density are associated with poorer clinical outcomes. However, screening for cancer‐related sarcopenia (typically defined as low muscle mass) is not routinely conducted. The European Working Group on Sarcopenia in Older People (EWGSOP) recommends an algorithm for sarcopenia screening and diagnosis in clinical settings, with sarcopenia based on muscle strength and mass, and severity on physical performance. We explored the application of the EWGSOP2 algorithm to assess sarcopenia in six ovarian cancer patients receiving neoadjuvant chemotherapy.

**Methods:**

We assessed sarcopenia risk with the SARC‐F screening questionnaire (at risk ≥4 points), muscle strength with a handgrip strength test (cut point <16 kg) and five times sit‐to‐stand test (cut point >15 s), muscle mass by skeletal muscle index (SMI in cm^2^/m^2^ from a single computed tomography [CT] image; cut point <38.5 cm^2^/m^2^), and physical performance with a 4‐m gait speed test (cut point ≤0.8 m/s).

**Results:**

Of six participants, none were identified as “at risk” for sarcopenia based on SARC‐F scores. Two participants were severely sarcopenic based on EWGSOP2 criteria (had low muscle strength, mass, and physical performance), and five participants were sarcopenic based on muscle mass only.

**Discussion:**

Ovarian cancer patients with low muscle mass during neoadjuvant chemotherapy may not be identified as sarcopenic based on the EWGSOP2 diagnostic algorithm. While lacking a universally accepted definition for cancer‐related sarcopenia and cancer‐specific recommendations for the screening, diagnosis, and treatment of sarcopenia, ovarian cancer clinicians should focus on the diagnosis and treatment of low muscle mass and density.

## 1 Introduction

**FIGURE 1:**
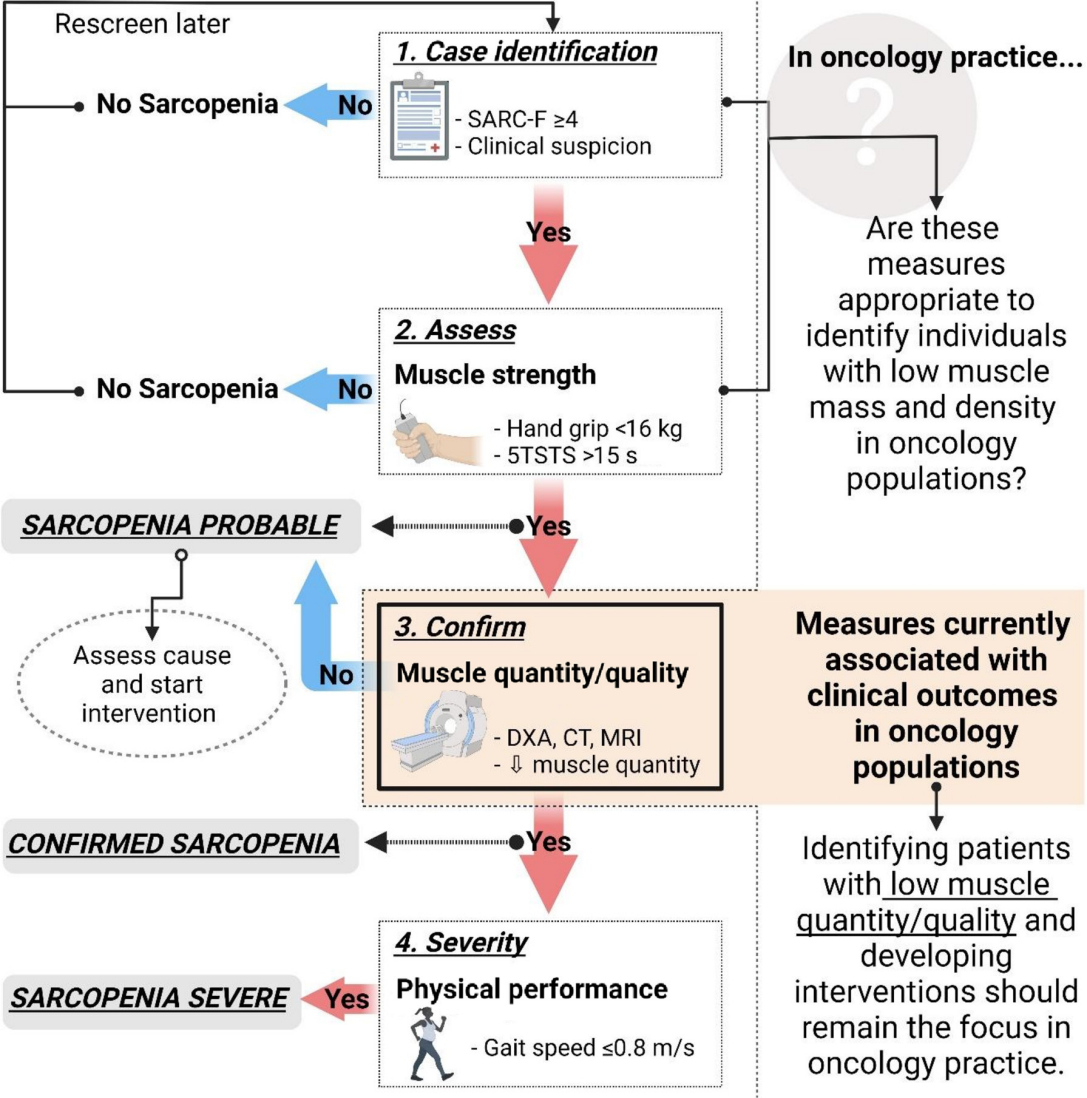
Challenges in applying an existing sarcopenia diagnostic algorithm European Working Group on Sarcopenia in Older People revised sarcopenia definition (EWGSOP2) in oncology practice. CT, computed tomography; DXA, dual‐energy x‐ray absorptiometry; MRI, Magnetic resonance imaging.

Sarcopenia is “a progressive and generalized skeletal muscle disorder” associated with an increased risk of physical disability and mortality [1]. Although sarcopenia is primarily a condition associated with aging, it is often related to secondary factors such as physical inactivity, malnutrition, and disease such as cancer [2]. Generally, both low muscle mass and low muscle function (low muscle strength and/or impaired physical function) are required for the definition of sarcopenia [1, 3, 4, 5, 6]. In 2018, the European Working Group on Sarcopenia in Older People (EWGSOP) published an updated consensus statement (EWGSOP2), indicating muscle strength, and not muscle mass, as the primary parameter to diagnose sarcopenia (EWGSOP2). The group recommends using their F–A–C–S (Find cases, Assess, Confirm, Severity) algorithm to screen for and diagnose sarcopenia in clinical practice [1] (Figure 1).

There is a large and growing body of research on sarcopenia in cancer populations. However, most publications include only low muscle mass in their definition of cancer‐related sarcopenia [7]. Low muscle mass is highly prevalent in people with cancer [7, 8] and has been associated with decreased quality of life and treatment tolerance [9, 10, 11] and increased mortality [12, 13]. Nonetheless, low muscle mass is not unique to sarcopenia. It is also a feature of malnutrition [14] and cachexia [15, 16], both common in cancer populations [16, 17]. It has been suggested that studies solely focusing on low muscle mass or muscle loss in cancer populations should use the term myopenia rather than sarcopenia [18]. In a recent systematic review of 226 papers, Couderc et al. [7] found that the inclusion of muscle strength and/or physical function measures reduced the prevalence of sarcopenia diagnoses in cancer patients and increased the predictive value for overall and progression‐free survival and postoperative complications. To date, most studies reporting on cancer‐related sarcopenia are retrospective in nature and few studies have incorporated measures of muscle function (i.e., muscle strength and/or physical function) in their sarcopenia assessment [18, 19]. Despite evidence that cancer‐related sarcopenia (defined as low muscle mass) is highly prevalent [7, 8] and has important prognostic value [9, 10, 11, 12, 13], routine screening for sarcopenia, either defined as low muscle mass, or as low muscle strength and mass, is rare in clinical cancer practice. Although several calls have been made for the early identification and treatment of cancer patients with sarcopenia in clinical practice [20, 21], diagnostic algorithms proposed for the screening and diagnosis of sarcopenia in geriatric populations, such as the EWGSOP2 algorithm, may not be suitable for cancer populations. In older adults without cancer, a decline in muscle strength is associated with muscle mass loss, with the age‐related loss of muscle strength occurring earlier than the associated loss of muscle mass [22]. In contrast, in cancer patients, the loss of muscle mass appears to be accelerated as a result of tumor‐driven systemic inflammation and metabolic disruptions [23] and may precede a measurable loss in muscle strength.

In ovarian cancer, sarcopenia (defined as low muscle mass and/or low muscle density assessed from computed tomography [CT] scans) is highly prevalent already at diagnosis, with further decreases in muscle mass during treatment [24]. A systematic review of 15 studies by Tranoulis et al. [25] found that 41% of ovarian cancer patients presented with low muscle mass. Sarcopenia in ovarian cancer is associated with an increased risk of postsurgical complications, chemotherapy toxicity, and mortality [25, 26, 27]. To our knowledge, no ovarian cancer study to date has included a screening tool, or strength or functional assessments to evaluate sarcopenia. Therefore, the aim of this study was to explore the application of the EWGSOP2 algorithm to assess sarcopenia in advanced‐stage ovarian cancer patients treated with neoadjuvant chemotherapy (NACT).

## 2 Methods

### 2.1 Setting and Participants

This case series of advanced‐stage epithelial ovarian cancer patients undergoing NACT at the St John of God Hospital, Subiaco, in Perth, Australia, were recruited between September 2019 and March 2021. Patients were eligible for inclusion if they: (1) had an abdominal CT scan undertaken after the second or third NACT cycle, and (2) were willing to complete questionnaires and undergo a muscle strength and functional assessment on the day prior to or of the third NACT cycle. Ethical approval was granted by the St John of God Health Care (Ref. no. 1542, 19/7/2019) and the Edith Cowan University Human Research Ethics Committees (Ref. no. 2019‐00629‐SCHOFIELD, 28/7/2019). All participants provided informed consent.

### 2.2 Data Collection

Participants were screened and assessed for sarcopenia according to the F–A–C–S algorithm [1] (Figure 1). Sarcopenia risk was determined with the SARC‐F questionnaire (a score of ≥4 points is considered predictive of sarcopenia) [28]. To diagnose probable sarcopenia, muscle strength was measured with a handgrip strength test (Jamar handgrip dynamometer, Lafayette Instrument Company, Inc., Lafayette, USA) and a five times sit‐to‐stand test (5TSTS). Diagnoses were based on cutoff values of <16 kg for grip strength and >15 s for 5TSTS [1]. Muscle mass (muscle quantity) was measured from CT scans routinely undertaken after two or three NACT cycles. For muscle mass, total abdominal muscle cross‐sectional area was measured from a single CT image at the third lumbar vertebral level and normalized for height (skeletal muscle index [SMI], cm^2^/m^2^) [29]. While EWGSOP2 recommends the use of various measurement tools, including CT, for muscle mass (muscle quantity), it currently only includes cutoff values for dual‐energy x‐ray absorptiometry (DXA). As such, the cut point of 38.5 cm^2^/m^2^ recommended by the Clinical Oncology Society of Australia (COSA) was applied [20, 29]. To determine the severity of sarcopenia, physical performance was measured with a 4‐m gait speed test. A gait speed of ≤0.8 m/s indicates severe sarcopenia [1].

## 3 Results

Of 18 eligible ovarian cancer patients approached to participate, eight were recruited. A complete sarcopenia assessment could be conducted for six patients, as CT scans could not be retrieved for two patients. The six participants had a mean age of 63 ± 11 years (range: 46–72) and a mean BMI of 24.1 ± 3.8 (Table 1).

**TABLE 1:** Patient characteristics and cancer stage.

Case	Age (years)	BMI (kg/m^2^)	Relationship status	Educational attainment	Smoking status	Number of comorbidities	Cancer stage
1	71	27.4	Partnered	Completed secondary school	Former	1	4A
2	71	28.4	Not partnered	University degree	Former	0	3C
3	66	18.4	Partnered	Postsecondary certificate/diploma	Former	0	3C
4	72	21.8	Partnered	Completed secondary school	Former	1	4A
5	53	22.7	Partnered	Completed secondary school	Current	0	3C
6	46	25.9	Partnered	University degree	Former	0	3C

### 3.1 Sarcopenia Assessment

**FIGURE 2:**
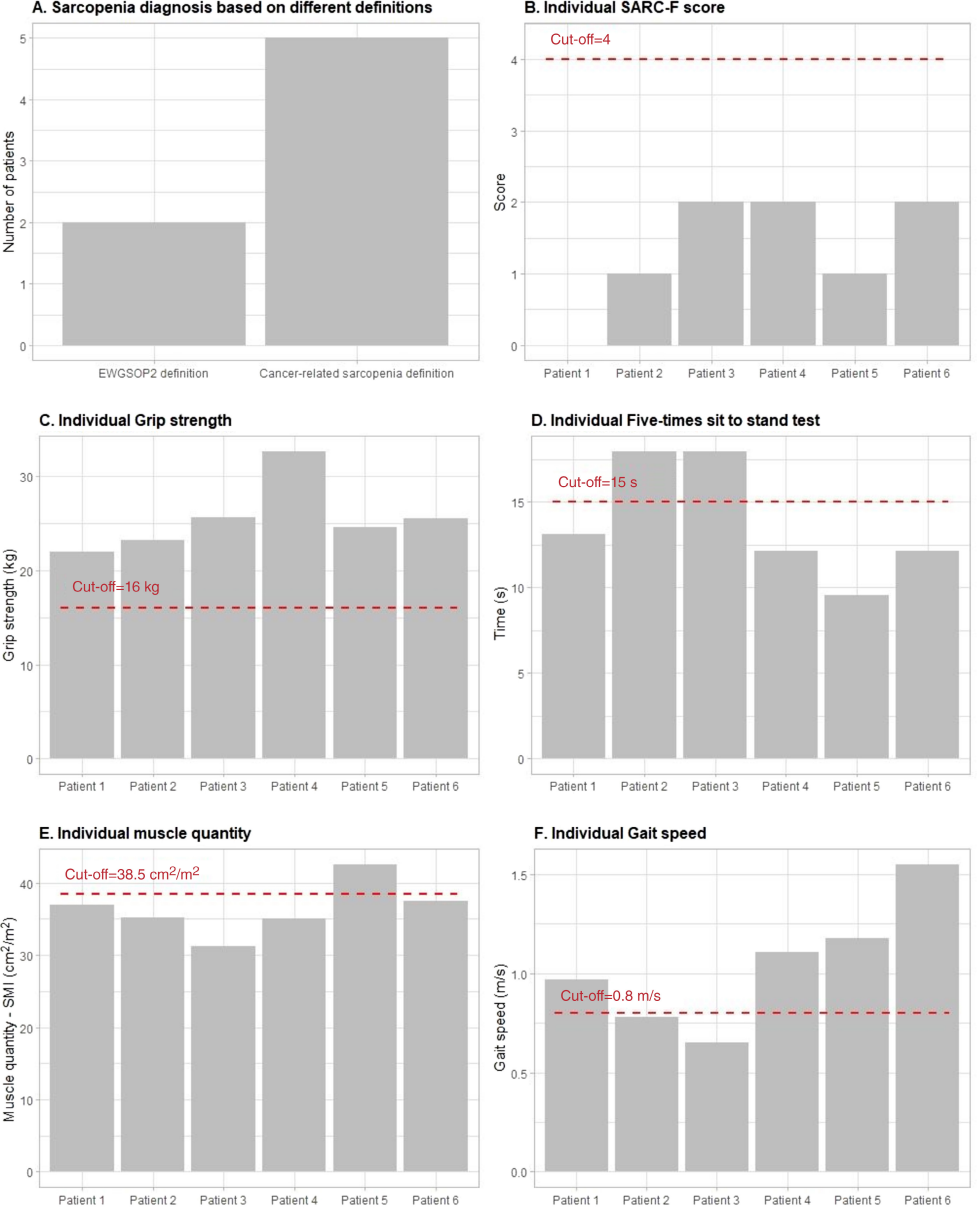
Results of the European Working Group on Sarcopenia in Older People revised sarcopenia definition (EWGSOP2) screening and assessment process. SMI, skeletal muscle index.

Results of the EWGSOP2 assessment process are presented in Figure 2. The SARC‐F scores ranged from 0 to 2. Mean handgrip strength was 25.6 ± 3.7 kg (range: 22.0–32.7) and mean 5TSTS time was 13.8 ± 3.4 s (range: 9.6–18.00). None of the participants scored below the recommended cut point of <16 kg for handgrip strength and two participants had a 5TSTS time slower than the 15‐s cut point. Mean SMI was 36.4 ± 3.7 cm^2^/m^2^ (range: 31.3–42.6). Five of the six participants (83%) had a SMI below the 38.5 cm^2^/m^2^ cut point. Mean gait speed was 1.0 ± 0.3 m/s (range: 0.7–1.6), with the same two participants who scored >15 s for 5TSTS achieving a slower time than the recommended 0.8 m/s.

Based on the EWGSOP2 algorithm, none of the participants were identified as “at risk” for sarcopenia based on SARC‐F scores and, as such, no further assessment would take place. However, two of the six participants presented with low muscle strength and mass, as well as low physical performance, indicating severe sarcopenia. In contrast, five of the six participants had low muscle mass and would be considered sarcopenic based on the currently accepted definition of cancer‐related sarcopenia.

## 4 Discussion

We examined the application of the EWGSOP2 algorithm to assess sarcopenia in a small sample of advanced‐stage ovarian cancer patients undergoing NACT. In this exploratory study, none of the women who had low muscle strength or low muscle mass during NACT were identified as at risk of sarcopenia during initial screening using the SARC‐F questionnaire. However, based on the EWGSOP2 sarcopenia definition of low muscle strength and mass, two of the six women were sarcopenic, while based on the current cancer‐related sarcopenia definition of low muscle mass, five of the six women were sarcopenic.

On initial screening with the SARC‐F, none of our participants were identified as at risk for sarcopenia. Both the EWGSOP2 and COSA recommendations stipulate that further sarcopenia assessment is only required for patients identified as “at risk” during screening. Therefore, no one in our sample would have been considered for further assessment. In a recent meta‐analysis, the SARC‐F, an inexpensive and easy‐to‐administer tool developed for rapid sarcopenia screening [28], was found to have low to moderate sensitivity (28.9%–55.3%) to detect sarcopenia when applying different diagnostic definitions [30]. SARC‐F scores correlate well with measures of muscle function in older adults with [31] and without cancer [28], but are not correlated with muscle mass [32, 33]. Thus, the SARC‐F questionnaire might be a better screening tool for low muscle function than low muscle mass, which could be an important limitation of using SARC‐F in cancer settings [34]. To our knowledge, no previous studies have investigated the utility of SARC‐F in ovarian cancer patients. Before recommendations can be made for the use of SARC‐F as a sarcopenia screening tool in this cancer group, a better understanding of the association between SARC‐F scores and the functional and structural components of sarcopenia is needed. It is worthwhile to note that, in contrast with the EWGSOP2 and COSA recommendations, the Asian Working Group for Sarcopenia (AWGS) 2019 algorithm does not require the screening of individuals with chronic medical conditions with SARC‐F or other recommended screening tools, but automatically qualifies them for assessment of possible sarcopenia with muscle strength or physical performance testing [4]. Models similar to the AWGS 2019 algorithm may be more appropriate for identifying sarcopenia in advanced‐stage ovarian cancer.

EWGSOP2 considers muscle strength the primary determinant of sarcopenia. In our sample, two participants presented with low muscle strength, or probable sarcopenia. According to the EWGSOP2 F–A–C–S algorithm, three of the five women in our sample who had low muscle mass, but not low muscle strength, would not have been diagnosed as sarcopenic. In contrast to our finding, Yee et al. [35] found the prevalence of probable sarcopenia (low muscle strength) in older adults to be more than three times higher than confirmed sarcopenia (low muscle strength and low muscle mass), supporting the notion that age‐related loss of muscle strength occurs earlier than the associated loss of muscle mass [22]. Muscle loss is a common feature of sarcopenia, but also of malnutrition and cachexia [21, 36]. It is possible that some of the women in our sample, all diagnosed with advanced ovarian cancer, experienced an accelerated loss of muscle mass as result of cancer‐related sequelae, such as systemic inflammation or malnutrition, that had not yet progressed to a loss of muscle strength measurable as probable sarcopenia. More research is needed to elucidate the relationship between cancer‐related muscle loss (irrespective of the cause) and loss of muscle strength.

To diagnose probable sarcopenia, EWGSOP2 recommends the use of a grip strength or chair stand test to measure muscle strength. An issue to consider in the clinical application of the EWGSOP2 algorithm is choice of muscle strength test. In our sample, two participants had low muscle strength based on the 5TSTS, while none had handgrip strength below the 16 kg cutoff value. It is unclear whether grip strength, 5TSTS, or a combination of both will better reflect the muscle strength component of sarcopenia in women with ovarian cancer. Further, if these tests are utilized during treatment and after treatment completion, the presence of peripheral neuropathy, a common side effect of chemotherapy treatment [37], may affect results of both tests and thus the diagnosis of probable sarcopenia.

The EWGSOP2 consensus statement stipulates the use of muscle quantity and/or muscle quality measures to confirm a diagnosis of probable sarcopenia, but does not provide a preferred definition of, or cutoff values for, muscle quality. In cancer research, low muscle quality or myosteatosis (decreased muscle density due to excess fat deposit measured from routine CT scans) and low muscle mass or cancer‐related sarcopenia are acknowledged as two distinct and independent muscle phenotypes [38, 39]. Additionally, low muscle density is more consistently associated with poorer clinical and patient‐reported outcomes than low muscle mass in cancer populations [18, 40]. This further challenges the application of the EWGSOP2 algorithm in oncology settings.

Our study has limitations worthy of comment. First, the case series included only six patients. Further, the homogeneous nature of our sample in terms of disease stage and treatment stage and type limits generalizability of our findings to other contexts (different treatments or time points). Nonetheless, this exploratory study is the first, to our knowledge, to apply the EWGSOP2 diagnostic algorithm, recommended for sarcopenia assessment in clinical practice, in advanced‐stage ovarian cancer patients. In women with ovarian cancer, low skeletal muscle mass and density are highly prevalent and are associated with worse treatment and survival outcomes [25, 26, 27]. Abdominal CT scans are routinely undertaken in ovarian cancer care for diagnostic and surveillance purposes and are available for the assessment of muscle mass and muscle density at different time points during and after ovarian cancer treatment. Based on our preliminary findings, healthcare professionals involved in ovarian cancer treatment and care should focus on the diagnosis and treatment of low muscle mass and density. Admittedly, challenges remain around the establishment of universally accepted cutoff values for low muscle mass and density, and further research is urgently needed in this regard.

## 5 Conclusion

We found that ovarian cancer patients who have low muscle mass during NACT were not identified as at risk of sarcopenia during initial screening using the EWGSOP2 algorithm. Based on our preliminary findings, the EWGSOP2 diagnostic algorithm may not be appropriate for identifying those with low muscle mass in ovarian cancer populations. Nevertheless, the findings need to be further substantiated in a larger cohort.

## Author Contributions

**Christelle Schofield:** conceptualization, investigation, writing – original draft, formal analysis, methodology, visualization, validation, writing – review and editing, project administration. **Dennis R. Taaffe:** writing – original draft, conceptualization, methodology, writing – review and editing, formal analysis, visualization, supervision. **Robert U. Newton:** conceptualization, writing – review and editing, supervision, methodology. **Daniel A. Galvão:** conceptualization, writing – review and editing, supervision. **Paul A. Cohen:** conceptualization, writing – review and editing, resources, supervision. **Jin‐Soo Kim:** writing – review and editing, visualization, formal analysis. **Tarek Meniawy:** resources, writing – review and editing, methodology, conceptualization. **Carolyn Peddle‐McIntyre:** methodology, conceptualization, writing – original draft, supervision, formal analysis, visualization, writing – review and editing.

## Conflicts of Interest

The authors declare no conflicts of interest.

## Data Availability

Deidentified data used and/or analyzed in this study are available from the corresponding author upon reasonable request.
